# The interplay between gastrointestinal dysfunction and gut microbiota dynamics in sepsis

**DOI:** 10.3389/fcimb.2026.1761536

**Published:** 2026-04-16

**Authors:** Lirong Zheng, Tingting Jia, Yang Li, Zhihao Zhang, He Su, Ruifen Zhang

**Affiliations:** 1 Second Affiliated Hospital of Tianjin University of Traditional Chinese Medicine, Tianjin, China; 2 The Second Clinical College of Guangzhou University of Chinese Medicine, Guangzhou, Guangdong, China; 3 Inner Mongolia Autonomous Region Hospital of Traditional Chinese Medicine, Hohhot, Inner Mongolia, China

**Keywords:** entericpathophysiology, gastrointestinal dysfunction, host–microbe interactions, microbiome-targeted therapies, mucosal immunity, sepsis, translational immunology

## Abstract

Sepsis frequently involves early gastrointestinal dysfunction, in which intestinal barrier breakdown and microbiota dysbiosis amplify systemic inflammation and contribute to multi-organ failure. Emerging evidence indicates that the gut is not merely a bystander in sepsis but an active driver of pathogenic cascades through epithelial injury, mucosal immune dysregulation, ischemia–reperfusion stress, and impaired motility, collectively promoting microbial translocation and immune deviation. In parallel, sepsis is associated with profound remodeling of the gut microbiome, characterized by reduced commensal diversity, expansion of pathobionts, and functional shifts in key microbial metabolites, including short-chain fatty acids, bile acids, and tryptophan-derived products, which further compromise mucosal integrity and host immune tone. This narrative review synthesizes experimental, translational, and clinical findings to elucidate the bidirectional interaction gut barrier–microbiota interplay in sepsis and to summarize mechanistic links across epithelial, immune, and metabolic signaling pathways, including gut-liver and gut-brain axes relevant to sepsis-associated organ dysfunction. dysfunctional microbial community leads to systemic immune deviation, multi-organ dysfunction and sepsis-associated encephalopathy, a common and severe neurological complication of sepsis. We also discuss emerging therapeutic strategies targeting the gut–microbiota axis—such as early enteral nutrition, prebiotics/postbiotics, defined microbial consortia, fecal microbiota transplantation, and metabolite-based supplementation—and evaluate their potential and limitations in septic populations. Finally, we highlight key challenges, including unresolved causality, inter-individual variability, context-dependent responses, and safety concerns, underscoring the need for longitudinal multi-omic profiling, host–microbiome phenotyping, and mechanism-informed interventional trials to enable precision microbiome-based approaches for sepsis.

## 1 Introduction

Sepsis is an inflammatory disorder caused by an infectious process that disrupts normal host functions and can cause organ dysfunction (Ackerman et al., 2021). Despite advances in antimicrobial therapy and organ-supportive care, sepsis remains a leading cause of mortality in intensive care units, and effective mechanism-based adjunctive therapies are still limited. Historically, in the medical community, the focus of the majority of research and clinical practice have been the lung, kidney, cardiovascular system, and vasculature for many years due to their apparent susceptibility to damage from the systemic inflammatory response. While clinicians were aware that the gastrointestinal (GI) tract could be damaged by hypotension, the use of vasopressors, and systemic inflammation, historically, the GI tract was typically viewed as being vulnerable rather than being a major contributor to the pathophysiology of sepsis. However, with increased understanding of the relationship between the GI tract and the host’s immune system, the GI tract is now considered a key area where barrier function, immune regulation, and the ecological balance of microorganisms interact to either exacerbate or decrease the effects of the systemic inflammatory response associated with sepsis (Di Vincenzo et al., 2024; Niu and Chen, 2021; Zhou and Liao, 2021). mportantly, accumulating evidence suggests that sepsis-associated GI dysfunction is not merely a consequence of systemic inflammation; rather, barrier failure and dysbiosis can actively amplify inflammatory signaling, promote microbial translocation, and propagate organ dysfunction. Based upon our current understanding of the relationships among these components, it is clear that both GI dysfunction and alterations in the composition of the GI tract’s microbiota have direct implications for the outcomes of critical illness both acutely and over the long term. Notably, the gut-brain axis, as a key cross-organ communication pathway, mediates the crosstalk between intestinal dysbiosis and central neuroinflammation, which is closely linked to the development of sepsis-associated encephalopathy.

Given the rapidly expanding but fragmented literature, the aim of this narrative review is to synthesize current evidence on the bidirectional interplay among gut microbiota alterations, intestinal barrier dysfunction, and immune dysregulation specifically in sepsis, and to discuss translational opportunities for targeting the gut–microbiota axis. The scope of this review includes: (i) the structure and immune layers of the intestinal barrier and the host–microbiota interface; (ii) sepsis-related drivers of barrier injury, including microcirculatory disturbance, hypoperfusion/ischemia–reperfusion stress, and immune imbalance; (iii) compositional and functional remodeling of the gut microbiome and key microbial metabolites; (iv) emerging therapeutic strategies and the strength of supporting evidence; and (v) limitations, challenges, and future research directions. We focus on sepsis, rather than critical illness in general, because sepsis represents a distinctive clinical context in which systemic inflammation and immune dysfunction coincide with profound antibiotic exposure, vasopressor use, hypoperfusion, and nutrition-related perturbations—factors that together accelerate ecological collapse of the gut microbiome and compromise epithelial integrity more dramatically and consistently than many other ICU syndromes. Accordingly, sepsis provides a clinically relevant framework to interrogate causality, timing, and safety of microbiome- and barrier-directed interventions.

### 1.1 Historical evolution of the gut–sepsis concept

In early clinical and experimental medical literature, the description of sepsis focused on the body’s overall reaction to infection (fever, low blood pressure, rapid heart rate, etc.) rather than the specific reactions occurring within individual organs or systems (such as the gastrointestinal system). In terms of the GI tract, it was primarily considered through obvious symptoms (ileus, gastrointestinal hemorrhage, mucosal ulceration) that arose from the effects of shock and reduced blood flow to the area, and thus the thought process centered on the fact that the intestine was simply an “innocent victim” of the systemic effects of shock and blood flow reduction; there was little attention to local processes in the intestine that would affect how the illness progressed (Chen et al., 2023; Wang et al., 2025).

As time passed and additional physiological measures were used to assess patients with sepsis, investigators demonstrated significant changes in mesenteric blood flow, mucosal oxygenation, and intestinal permeability in both animal models and human subjects. Mucosal biopsy techniques, permeability assays, and regional perfusion imaging also documented the sensitivity of the intestinal mucosa to even small disruptions in its microvascular circulation (Chang et al., 2022; Doudakmanis et al., 2021) This information established the theory that the intestinal mucosa acts as a “driver” for systemic inflammation via passage of luminal bacteria, endotoxin, and other microbial products across a damaged epithelial barrier into the circulatory system. While the focus remained mostly on structural damage and loss of the integrity of the intestinal epithelium, this theory moved the GI tract closer to being at the center of sepsis’ pathophysiologic processes (Ji et al., 2023; Schäfer et al., 2022).

Culture-independent microbiological techniques as well as high-throughput DNA sequencing have again dramatically changed our understanding of disease states. We now realize that critical illness and sepsis is not just due to an overgrowth of a few pathogens; however, it represents an extreme ecological disruption of complex microbial ecosystems. These studies show that septic patients have reduced diversity of their intestinal microbiota; lose beneficial commensal microorganisms; and have increased levels of opportunistic bacteria that are adapted to survive in hospitals and the inflamed intestine (Park et al., 2024; Weiss et al., 2021). When we integrated the recent microbiota data into prior research on barrier function and injury, we obtained a far more detailed view of how gastrointestinal dysfunction occurs in sepsis; namely through structural damage to the mucosal lining (the “barrier”) and ecological collapse of the microbial community within the GI tract, and that these two processes interacted dynamically with one another in a reciprocal or bidirectional manner.

### 1.2 Physiology of the gastrointestinal barrier and host–microbiota interface

The intestinal wall acts as an important multi-layered protective mechanism to protect the densely populated intestinal lumen from the inside environment (sterile), while also allowing for nutrient absorption and immune surveillance (Gieryńska et al., 2022; Okumura and Takeda, 2025). While the most visible part of this complex is a single layer of rapidly renewing epithelial cells that are held together by tight junctions, it also includes specialized epithelial cells (such as goblet cells, Paneth cells, and enteroendocrine cells) which help in providing mucosal protection and serve as an interface between the immune system and both the nervous and endocrine systems (Barbara et al., 2021; Di Sabatino et al., 2023). In addition, these tight junctions allow control of the paracellular flow of water, ions, and macromolecules; they are composed of transmembrane proteins and scaffold proteins. The epithelial surface is covered with mucus that protects by physically and biochemically acting as a barrier rich in glycoproteins and antimicrobial peptides, especially in the colon, the area of the intestine that has the greatest amount of microbes (Acat and Yildirim, Seo et al., 2021; Song et al., 2023).

Beneath the epithelial layer lies the lamina propria, which contains many types of immune cells, including macrophages, dendritic cells, innate lymphoid cells, and lymphocytes. Collectively these cells survey for antigens in the lumen and respond to all microbial-related signals. Immune cells have regulatory mechanisms to limit their response to non-pathogenic (commensal) microorganisms and non-digestive food substances in order to allow for rapid responses to pathogenic microorganisms. The immune cell function is also regulated by local and systemic signals such as those generated by the enteric nervous system, local neurotransmitters, and circulating hormones that regulate motility, secretion, and blood flow through different areas of the GI tract. Therefore, the GI tract acts as an immune/neuroendocrine unit or organ rather than just as a single tube for digestion (Aguilera-Lizarraga et al., 2022; Bildstein et al., 2024).

The gut microbiome is located within the structural and immunologic environment that contains it. The gut microbiome is an extremely large and metabolically active ecosystem. The various microbial populations in the gut microbiome help to digest complex carbohydrate structures into simple structures through enzymatic activity, produce short-chain fatty acid from non-digestible carbohydrates, transform primary bile acids into secondary bile acids, and metabolize amino acids and other substances, including those that are toxic (xenobiotic) to humans (Flint et al., 2012). Many of the products of microbial metabolic processes improve the strength of the barrier function of the intestinal mucosa, provide nutrients for the enterocytes, and direct the differentiation of the cells of the immune system (Fan et al., 2022). Additionally, the microbiota provides a mechanism to inhibit the colonization of the gut by invasive pathogens through occupation of available ecological niches and the synthesis of antimicrobial compounds. Under healthy conditions, the physiology of the intestine and the ecology of the microbiota exist as a dynamic interplay between the host tissues and the microbial populations, where both are mutually maintaining the barrier integrity of the intestinal mucosa and the homeostasis of the immune system.

### 1.3 Sepsis-induced gastrointestinal dysfunction

Systemically acting inflammatory mediators, sympathetic stimulation, and endothelial injury damage the microvascular circulation in the mesentery, which reduces blood flow and leads to patchy hypoperfusion and localized ischemia in the intestines (Lu et al., 2025a; Pulido and Ehrenpreis, 2021; Raia and Zafrani, 2022). As a result of these alterations, the small intestine has a significantly increased risk for injury because it has high oxygen demands; therefore, it is dependent on a well-oxygenated stem cell niche located at the base of each intestinal crypt to repair itself. The subsequent occurrence of ischemia-reperfusion injuries results in loss of enterocytes (epithelial cells) through apoptosis, atrophied villi; and compromised architecture of tight junctions that separate enterocytes (Deng et al., 2022; Li et al., 2022; Wang et al., 2024). These changes in number and function of enterocytes also compromise the protective function of the enterocyte layer and expose deeper layers of the enterocyte to luminal content (Krstić Ristivojević et al., 2021).

Sepsis also has a profound impact on intestinal movement (motility). Several factors influence gastrointestinal motility during sepsis. These include alterations in the enteric nervous system, the action of inflammatory mediators on smooth muscle, and the effect of pain management medications such as morphine and sedatives on smooth muscle. The degree of alteration in gastrointestinal motility can vary depending upon the extent of these influences (Castro et al., 2023; Docsa et al., 2022; Doudakmanis et al., 2021).

In addition to contributing to the degree of alteration in gastrointestinal motility, the prolonged contact time between microorganisms found in the lumen of the small bowel with the mucosa, altered oxygen gradients throughout the gut, and altered availability of nutrients contribute to an altered ecological environment in the gut. In addition to creating an ideal environment for some microorganisms to grow and multiply, luminal stasis increases the likelihood of translocation of those organisms across the epithelial layer into the bloodstream (Guilliams and Drake, 2021; Ren et al., 2025).

Additional mechanical forces generated by splanchnic edema and increased intra-abdominal pressures create additional barriers to both perfusion and movement of the intestines. The structural and functional changes associated with sepsis result in clinical manifestations of gastrointestinal dysfunction characterized by feeding intolerance, abdominal distension, delayed gastric emptying, and mucosal bleeding or stress ulcer formation (Eaton and Faulds, 2024; Górski and Swidnicka-Siergiejko, 2024; Reintam Blaser et al., 2022). However, from a mechanistic standpoint, the gastrointestinal dysfunction observed in sepsis is more than just symptoms; it is a state where the barrier function of the gut, immune regulatory functions, and the ability to contain harmful microorganisms have been significantly compromised. As a result, rather than functioning as a controlled entry point, the gut becomes a potential source of pro-inflammatory and microbial signals that can exacerbate and amplify the systemic response characteristic of sepsis (Nie et al., 2024; Stepanova, 2022).

### 1.4 Gut microbiota reconfiguration during critical illness and sepsis

The gut microbiome is significantly altered during a critical illness or sepsis. There are many factors involved which lead to a complete reversal of what was once an equilibrium ecosystem. The use of broad-spectrum antibiotics will eliminate many of the more susceptible bacterial populations (obligate anaerobes), which produce many of the beneficial metabolic by-products, including short-chain fatty acids. The amount of feed provided through the enteral route, the amount of nutrients supplied via total parenteral nutrition, and the amount of fermentable substrates available for the commensals to grow are all limited. Additionally, increased oxygenation in the gut lumen due to increased inflammatory processes and decreased integrity of the intestinal epithelium provide facultative aerobic bacteria and organisms that grow well in inflamed tissues with a competitive advantage (Özdirik et al., 2021).

The overall outcome of the changes to the intestinal flora results in a pattern of reduced biodiversity, a loss of what are called “core” commensals (those organisms which are typically present), and an increase in opportunistic bacteria, fungi, and sometimes viruses. This has been referred to as dysbiosis; however, each patient will have their own unique microbial community profile. Many common elements exist across all such communities. These include the decreased presence of organisms contributing to the maintenance of the epithelial barrier and/or immunological tolerance and an increased prevalence of those organisms capable of promoting nosocomial infections or drug resistant strains (Philips et al., 2022). The metabolic capabilities of the microbiota are also altered: there is a decrease in the generation of products of the microbiota which contribute to maintaining the integrity of the epithelial barrier, and there is an increase in the accumulation of products which may stimulate inflammation or are toxic to the host. There are also alterations in the conversion of bile acids and in tryptophan metabolism, which can further alter host signaling pathways in the liver, brain and immune system (Sun et al., 2023; Wasyluk and Zwolak, 2021).

The ecological modifications that occur as a result of these tissue level events do not happen in an isolated manner. The continuously inflamed and compromised epithelial layer and the continuously inflamed lamina propria are continually being exposed to the new shapes of the microbial communities and their products. Therefore, the microbial molecular patterns (PAMPs) will continue to stimulate cytokines through interaction with the PRRs found on epithelial and immune cells and further disrupt the damaged epithelial layer (Cicchinelli et al., 2024). Thus, a dysbiotic microbiota can be considered to create a self-reinforcing cycle between the environmental and tissue injury by contributing to the same type of injuries that contribute to its proliferation and growth. Therefore, reconfiguration of the microbiota can be seen as both a result of the disturbances caused by sepsis and a cause for continued progression of those disturbances.

## 2 Pathophysiology of gastrointestinal dysfunction in sepsis

The gastrointestinal system’s malfunction during sepsis occurs through the convergence of several factors, including mesenteric microcirculatory failure, epithelial barrier injury, dysregulated neuromuscular control, and impaired mucosal immune homeostasis (Jacobi, 2022). In sepsis, these insults are often intensified by infection-driven systemic inflammation, hemodynamic instability, vasopressor exposure, and antibiotic-associated ecological disruption, collectively predisposing the gut to barrier failure and dysbiosis. The small intestine does not experience a single type of insult; rather, it experiences multiple insults in a patchwork or mosaic pattern, shaped by the infection source, host premorbid state, and the intensity of organ support (Raia and Zafrani, 2022; Tang et al., 2022). During the early stages of sepsis, systemic inflammation and hemodynamic instability impair mesenteric perfusion, resulting in ischemia–reperfusion injury and microvascular leakage that prime epithelial and immune compartments for structural and functional breakdown (Lian et al., 2024; Zhang et al., 2024). Disruption of tight junction complexes leads to disorganized junctional assembly, mucus layer thinning or irregularity, and abnormal epithelial turnover with increased apoptosis and impaired regeneration. Simultaneously, sepsis-associated motility impairment promotes luminal stasis and alters oxygen and nutrient gradients that shape microbial niche conditions. Collectively, these changes reduce barrier containment and facilitate microbial translocation, thereby sustaining systemic inflammation and organ dysfunction in a self-reinforcing loop. To avoid redundancy with subsequent sections, this chapter focuses on sepsis-driven host mechanisms, while the sepsis-specific microbiome changes (composition, function, and clinical correlates) are detailed in the following dedicated section.

### 2.1 Mesenteric microcirculation and ischemia–reperfusion injury

Because the intestinal mucosa has a high dependence on oxygen and nutrient delivery to sustain rapid epithelial renewal, sepsis-related systemic vasodilation, relative hypovolemia, and reduced effective cardiac output can compromise mesenteric perfusion despite efforts to maintain flow to vital organs via neurohumoral compensation (Pecchiari et al., 2021; Valeanu et al., 2021) Therefore, the mesenteric circulation can respond in a complicated and often detrimental way: even when macro-hemodynamic targets appear adequate, microvascular flow becomes heterogeneous with segmental arteriolar constriction and capillary shunting, producing adjacent regions of hypoxia and relative preservation (Valeanu et al., 2021).

In addition, endothelial activation, leukocyte adhesion, and microthrombi further narrow or occlude micro vessels, promoting ischemia–reperfusion cycles and generation of ROS/RNS within the mucosa. ROS/RNS injure cellular membranes, mitochondria, and DNA, inducing epithelial apoptosis/necrosis—particularly at villus tips—thereby exposing the lamina propria to luminal contents and perpetuating inflammatory injury (Zhang et al., 2024). Over time, these processes lead to villous shortening, crypt deformation, and reduced absorptive and barrier surface area. While early microcirculatory impairment may manifest subtly (feeding intolerance, mild distension, altered bowel sounds), sustained regional ischemia can markedly restrict oxygen/nutrient delivery and limit drug distribution required for mucosal repair. Importantly, sepsis-associated microvascular dysfunction also alters luminal redox state and oxygen tension, favoring facultative anaerobes over obligate anaerobes, thereby linking perfusion failure directly to ecological remodeling. Thus, mesenteric microcirculatory failure initiates two intertwined trajectories: mucosal injury and a reshaped microbial niche.

### 2.2 Disruption of epithelial barrier architecture and tight junction integrity

The epithelial monolayer and tight junctions form the primary barrier between the host and the dense luminal microbial community. Under basal conditions, tight junctions are dynamic structures regulated by mechanical cues, cytokines, and growth factors; however, in sepsis, systemic and local pro-inflammatory mediators (e.g., TNF-α, IL-1β/IL-6, and chemokines) rapidly disrupt these regulatory pathways. These mediators alter intracellular signaling and cytoskeletal coupling, promoting tight junction protein endocytosis/translocation and degradation, thereby widening paracellular spaces and increasing permeability (Barbara et al., 2021; Paradis et al., 2021; Semin et al., 2021). As a result of these alterations, there is an increase in the size of paracellular spaces and thus increased permeability of the epithelial layer to water, ions, and larger molecules.

Inflammation and hypoxia also disrupt crypt stem-cell signaling (e.g., Wnt/Notch), tipping the balance toward apoptosis/necrosis at villus tips and inadequate regeneration, resulting in discontinuities over the damaged lamina propria (Chen et al., 2021; Sphyris et al., 2021). Similarly, Paneth and goblet cell dysfunction reduces antimicrobial peptides and mucus production, creating patchy, nonadherent mucus that increases epithelial exposure to microbial products.

**Figure 1:**
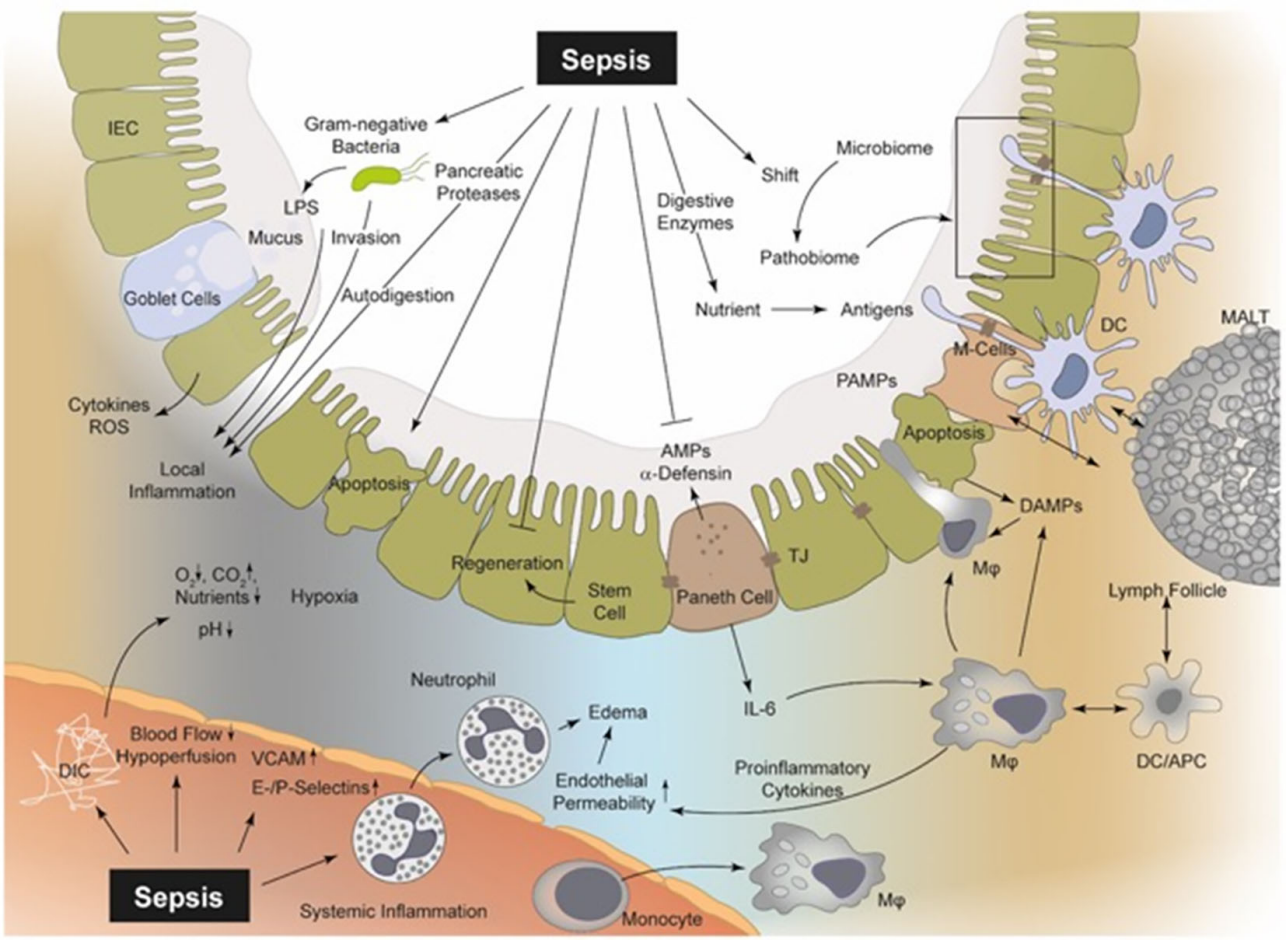
Sepsis is involved in several pathophysiological processes regarding the intestinal epithelial integrity, perfusion, coagulation, enzymatic response, and MIS. In sepsis, bacteria and their products (PAMPs), including LPS, PG, and bacterial DNA, can be recognized by PRRs (e.g., TLR2 and TLR4) upon the surface of macrophages, neutrophils, DCs, and even IECs. Thereby, intestinal macrophages and DCs as part of the MIS can detect luminal PAMPs via transepithelial dendrites (TEDs). Consequently, PAMPs induce a “cytokine storm” of pro-inflammatory mediators, which drive the local intestinal and systemic inflammation. The released mediators can lead to an upregulation of endothelial adhesion molecules (e.g., ICAM, VCAM, E-, and P-selectin), resulting in increased recruitment of neutrophils and monocytes and in turn to increased levels of pro-inflammatory cytokines and ROS. These cellular responses aggravate vasodilatation and induce a high level of capillary leakage with the development of interstitial edema. Local DIC is frequently observed during sepsis with a decreased supply of oxygen and nutrients, but increased carbon dioxide concentration. Hypoxia in turn leads to increased apoptosis and necrosis of IECs and the regeneration of these IECs is suppressed during sepsis. Furthermore, the IEC integrity is disrupted and bacterial translocation may be facilitated. Pancreatic proteases are capable of autodigestion and potentiation of MOF and self-digestion leads to an increased release of further DAMPs. MIS, mucosal immune system; PAMPs, pathogen-associated molecular patterns; DAMPs, danger-associated molecular patterns; LPS, lipopolysaccharide; PG, proteoglycan; PRR, pattern-recognition-receptors; TLR, toll-like receptor; DCs, dendritic cells; IECs, intestinal epithelial cells; TEDs, transepithelial dendrites; DIC, disseminated-intravascular-coagulation; MOF, multi-organ failure; ICAM, intercellular adhesion molecule 1; VCAM, vascular cell adhesion protein 1; ROS, reactive oxygen species; M-cells, microfold cells; AMPs, antimicrobial peptides; APC, antigen presenting cells; TJ, tight junctions; MALT, mucosa-associated-molecular pattern (Haussner et al., 2019).

When the barrier breaks down, increased passage of microbial components (LPS, peptidoglycan, and other MAMPs) activates PRRs on epithelial and immune cells, amplifying local and systemic inflammation (Cicchinelli et al., 2024; Yuki and Koutsogiannaki, 2021). Live bacteria may translocate into the lamina propria and beyond, potentially seeding portal/systemic circulation and increasing bacteremia risk (Rogers et al., 2023). In extreme cases, the translocation of bacteria can seed the portal and systemic circulations, thereby increasing the risk of bacteremia and distal tissue infections (Serek and Oleksy-Wawrzyniak, 2021). Barrier failure also exposes subepithelial nerves and vasculature to luminal irritants, further perturbing motility and vascular responses (Di Sabatino et al., 2023; Schäfer et al., 2022). Therefore, barrier disruption in sepsis transforms the intestinal surface from a regulated interface into a permeable and reactive site for host–microbe interaction. **Figure 1** illustrates the sequential development of barrier disruption, epithelial injury, tight junction destruction, mucus thinning, and bacterial translocation during sepsis-induced barrier disruption (Haussner et al., 2019).

### 2.3 Dysregulation of motility, luminal transit, and intestinal secretory function

Motility coordinates luminal mixing, nutrient absorption, and prevents prolonged residence of distal microbes in the small intestine (Dalziel et al., 2021; Ma et al., 2023; Waclawiková et al., 2022). During sepsis, motility is disrupted by inflammatory mediators, oxidative stress, altered enteric neurotransmission, and ICU medications (opioids, sedatives, catecholamines) (Liu et al., 2023b). Consequently, sepsis can cause ileus with delayed gastric emptying and prolonged transit time, which extends beyond feeding intolerance by reshaping substrate availability, pH/osmolarity, and spatial colonization. Consequently, sepsis can cause ileus with delayed gastric emptying and prolonged transit time, which extends beyond feeding intolerance by reshaping substrate availability, pH/osmolarity, and spatial colonization (Yang et al., 2021;. Quigley, 2023). Luminal stagnation increases opportunities for proximal colonization and biofilm formation and impairs clearance of mucus, sloughed cells, and exudate, increasing inflammatory mediator and microbial product exposure at the epithelial surface (Liu et al., 2023a).

Sepsis also alters secretory/absorptive processes via changes in transporters, ion channels, bile flow, and pancreatic secretions, thereby modifying enzyme and bile salt environments that influence microbial viability, adhesion, and metabolism (Liu et al., 2023a). Thus, sepsis-induced motility and secretory dysfunction create a new physicochemical milieu that shapes microbial behavior and barrier stress.

### 2.4 Mucosal immune responses and neuroenteric regulation

The intestinal mucosa is comprised of large numbers of immune cells that continually assess information from both the microbiome and host (Ahrend et al., 2025; Gustafsson and Johansson, 2022; Zhao and Maynard, 2022). In sepsis, heightened exposure to microbial metabolites and DAMPs can trigger robust innate activation via PRR upregulation, cytokine/chemokine induction, and leukocyte recruitment, which may secondarily injure the epithelial barrier (Zhou et al., 2021). Over time, mucosal immunity may transition toward dysfunction, including depletion/impairment of regulatory T cells and tolerogenic dendritic cells, limiting resolution and perpetuating epithelial injury (Ness et al., 2021). Adaptive immunity may also be compromised (reduced IgA and B-cell function), weakening immune exclusion; Paneth cell antimicrobial peptide production may decline, further altering microbiota composition (Cui et al., 2023).

Neuro-enteric regulation is also disrupted: inflammatory/ischemic insults impair ENS–vagal communication and anti-inflammatory reflexes (including cholinergic pathways), while stress hormones and sympathetic activation favor barrier-disruptive states (Holland et al., 2021). Thus, in sepsis, immune and neural signals often amplify—rather than resolve—tissue injury and ecological disruption.

Therefore, the pathophysiology of gastrointestinal dysfunction in sepsis involves an integrated network of microcirculatory impairment, epithelial barrier breakdown, motility/secretory abnormalities, and dysregulated mucosal immune–neural control.

These host-driven changes create selective pressures that remodel microbial composition and function, and the resulting dysbiosis can further worsen barrier integrity and systemic inflammation. The subsequent section will describe how the gut microbiota adapt to the septic milieu and how the changes in its composition and function feed back into the developing picture of gastrointestinal and systemic dysfunction.

## 3 Gut microbiota alterations in sepsis

Microbial populations in the gut and their functions undergo a drastic transformation as a result of sepsis—in many cases, just hours or days after a critical illness begins. The changes in gut microbiota due to sepsis occur due to an interplay between the effects of systemic inflammation, changes in blood flow to organs (i.e., organ perfusion), reduced gastrointestinal motility, dietary modifications, and widespread use of antibiotics (Liu et al., 2023a). Typically, healthy humans have an intestinal microbiota that is rich in diversity with complex relationships between different microbial species and has a large number of obligate anaerobic bacteria (Sun et al., 2023). Obligate anaerobic bacteria help maintain intestinal barrier function and promote immune system tolerance. However, when individuals develop sepsis, these relationships become disorganized, and the previously complex relationship of species collapses into a significantly less diverse and less organized group of microbial species.

### 3.1 Baseline ecological organization and early disruption during critical illness

Physiologically, gut bacteria are organized into spatial niches along the GI tract and across microenvironments, including the mucus-adjacent mucosa and the luminal compartment. Along the proximal–distal axis, bacterial density increases while oxygen tension generally decreases; nutrient availability also varies by site of digestion and absorption. Microbes interact through cross-feeding networks in which metabolites produced by one taxon serve as substrates for others, supporting a continuous supply of SCFAs, vitamins, and immunomodulatory compounds that maintain epithelial health and immune homeostasis (Rosa-Rizzotto et al.),. Host factors such as bile acids, antimicrobial peptides, and mucins further shape microbial composition and function.

Although sepsis-associated microbiota alterations can be detected early, sometimes preceding overt GI symptoms, they evolve in parallel with the development of barrier dysfunction described above and should be interpreted within a dynamic host–microbe process rather than as a single downstream consequence. Therefore, microbiota alterations in sepsis reflect both responses to host injury (hypoperfusion, inflammation, dysmotility) and active contributors that can further impair barrier repair and immune regulation. Additionally, as critical illness continues to evolve, the effects of the original perturbation of the microbiota become increasingly evident, ultimately producing a characteristic pattern of dysbiosis. Accordingly, we describe below a sepsis-centered dysbiosis pattern and its clinical relevance, while noting that ICU exposures (e.g., antibiotics, sedation, nutrition) interact with sepsis biology and must be accounted for in interpretation.

It is worth noting that antibiotics, while driving gut dysbiosis by eliminating beneficial obligate anaerobes, may also exert a transient protective effect in early sepsis: their microbial killing activity can temporarily reduce the load of pathogenic bacteria in the intestinal lumen, thereby mitigating bacterial translocation and attenuating the initial systemic inflammatory burst (McDonnell et al., 2021; Bai et al., 2025).However, this potential short-term benefit is counterbalanced by sustained ecological harm (loss of anaerobes, reduced colonization resistance, selection for resistant organisms), highlighting the need to evaluate antibiotic timing, spectrum, and duration in sepsis-specific microbiome studies.

### 3.2 Characteristic patterns of dysbiosis in sepsis

**Figure 2:**
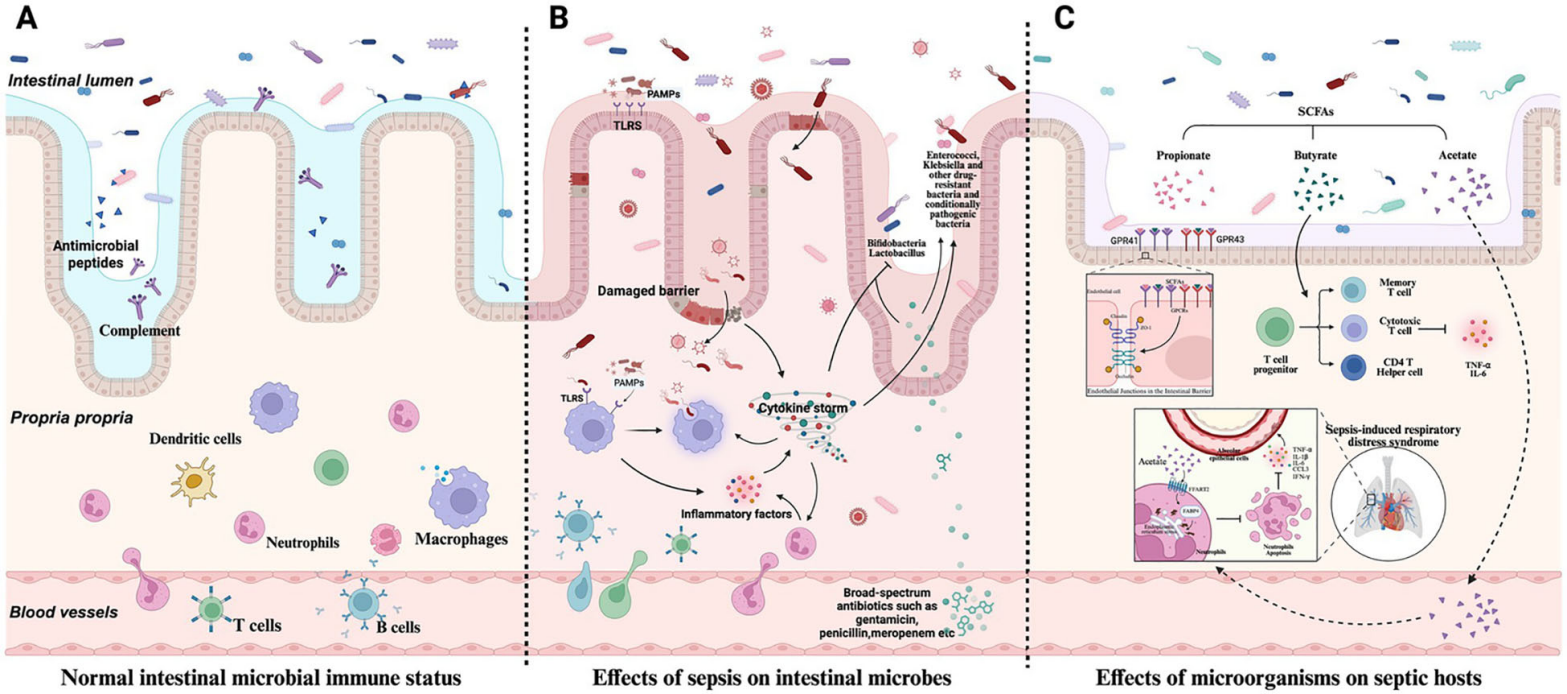
Host microbiome interactions in sepsis. **(A)** Under normal conditions, the host relies on innate immunity mechanisms (such as antimicrobial peptide secretion, complement activation, etc.) and adaptive immunity (such as antibody production, immune cell-mediated killing, etc.) to maintain the balance of intestinal microbial community. **(B)** The strong immune response triggered by sepsis breaks the original ecological balance of intestinal microbial community. **(C)** Microorganisms influence sepsis development and host immune response (Lu et al., 2025b).

Dysbiosis lacks a single standardized diagnostic criterion in sepsis and is most commonly described as reduced microbial richness/evenness, disrupted commensal–pathobiont balance, and functional instability beyond normal physiological fluctuation (Piccioni et al., 2024; Tao et al., 2024). While each patient may exhibit a distinct microbiota profile, shared features across septic cohorts include decreased alpha diversity, depletion of obligate anaerobes, and enrichment of facultative anaerobes/aerobes adapted to inflammatory and hospital environments (Liu et al., 2023a). Microorganisms that support barrier function and immune tolerance (e.g., butyrate-producing taxa and taxa linked to regulatory T-cell induction) often decline, whereas opportunistic or antibiotic-tolerant pathobionts increase. These compositional shifts frequently align with organisms implicated in nosocomial infections (e.g., Enterococcus, Enterobacterales, Staphylococcus) (Weiss et al., 2021). In some patients, dysbiosis progresses to “monodominance,” in which one or two taxa comprise most of the community, suggesting ecological collapse under strong selection pressure (commonly antibiotics and inflammation). In parallel, reduced motility and altered luminal conditions may promote proximal small-intestinal colonization by colonic-type bacteria, disrupting nutrient competition and mucosal immune signaling and potentially increasing microbial translocation risk. **Figure 2** shows host microbiome interactions in sepsis (Lu et al., 2025b).

These dysbiosis patterns are clinically relevant because reduced diversity and expansion of pathobionts have been associated with higher rates of secondary infection, prolonged organ dysfunction, and increased mortality in septic/critically ill cohorts (Iacob and Iacob, 2019). Where available, future revisions should prioritize sepsis-specific cohort studies and longitudinal sampling to strengthen these associations and reduce confounding by ICU exposures. The major characteristics of sepsis-related dysbiosis are outlined in **Table 1** as a summary of the most common compositional and functional changes associated with this condition and as a basis for the mechanisms related to this subject discussed further on.

**Table 1:** Characteristic features of gut microbiota dysbiosis in sepsis and their putative implications.

Domain	Typical change in sepsis	Mechanistic implication for gut environment	Potential systemic consequence	Specific examples of altered taxa
Overall diversity (Yin et al., 2019)	Marked reduction in species richness and evenness	Loss of ecological redundancy and resilience; collapse of cross-feeding networks	Greater vulnerability to pathogen overgrowth and colonization	Generalized loss of multiple commensal genera.
Dominant taxa (Yang et al., 2021)	Expansion of facultative anaerobes and hospital-adapted pathobionts	Enhanced tolerance to oxidative stress and antibiotics; biofilm formation	Increased risk of bloodstream infection and nosocomial pneumonia	Increase: *Enterococcus* spp. (e.g., *E. faecium*), *Enterobacteriaceae* (e.g., *E. coli, Klebsiella* spp.), *Staphylococcus* spp.
Beneficial anaerobes (Kullberg et al., 2025)	Depletion of butyrate-producing and mucosa-supporting commensals	Reduced short-chain fatty acid availability; impaired epithelial energy and repair	Weakened barrier integrity; exaggerated systemic inflammation	Decrease: *Faecalibacterium prausnitzii* (key butyrate producer), *Roseburia* spp., *Eubacterium rectale, Bifidobacterium* spp.
Small intestinal colonization (Fay et al., 2017)	Proximal overgrowth by colonic-type bacteria	Altered nutrient competition and local pH; increased exposure of proximal mucosa to toxins	Malabsorption, feed intolerance, and enhanced microbial translocation	Colonization of duodenum/jejunum by typical colonic flora (e.g., *Bacteroides* spp., *Enterobacteriaceae*).
Microbial metabolite profile (Beloborodova et al., 2018)	Shift away from trophic metabolites toward pro-inflammatory derivatives	Accumulation of metabolites that activate inflammatory pathways or impair mitochondrial function	Mitochondrial dysfunction, organ injury, and altered immunometabolism	Decreased: Butyrate, acetate. Increased: Succinate, p-cresol sulfate, certain deconjugated bile acids.
Antimicrobial resistance genes (Costa, 2008)	Enrichment of multidrug-resistant strains and resistance determinants	Limited effectiveness of standard antibiotic regimens; persistence despite therapy	Difficult-to-treat infections and increased mortality risk	Carriage of extended-spectrum beta-lactamase (ESBL) genes, vancomycin resistance genes (*vanA/vanB*), carbapenemase genes.

### 3.3 Functional and metabolic consequences of septic dysbiosis

Beyond compositional changes, sepsis-associated dysbiosis is characterized by functional remodeling of microbial metabolism and host–microbe signaling. One consistent observation is reduced production of SCFAs—particularly butyrate—which serves as a major energy source for colonocytes and a regulator of inflammatory signaling (Sun et al., 2023). Reduced butyrate availability can impair epithelial energy homeostasis and barrier repair processes, including tight junction maintenance, mucin production, and wound healing. Alterations in acetate and propionate may further influence systemic energy utilization and immune responses, collectively weakening microbiota-supported tolerance (Kim, 2023; Luu et al., 2020).

Dysbiotic communities may also show reduced biosynthesis of vitamins and cofactors and altered redox-related functions, potentially affecting mitochondrial function and DNA synthesis. In addition, dysbiosis can increase production/accumulation of potentially harmful metabolites (e.g., phenols, sulfur-containing compounds), which may impair mitochondrial respiration, injure endothelial cells, and promote pro-thrombotic tendencies (McBeth et al., 2025; Mostafavi Abdolmaleky and Zhou, 2024). These toxic compounds can either directly or indirectly inhibit mitochondrial respiration, cause damage to endothelial cells, increase the propensity for blood clot formation, and therefore potentially lead to systemic complications. Overall, the combination of all of these metabolic changes transforms the microbiota from a beneficial community that produces helpful regulatory substances into a detrimental community producing signals that will exacerbate inflammation, promote vascular dysfunction, and induce tissue injury.

## 4 Bidirectional interactions between gastrointestinal dysfunction and gut microbiota in sepsis

The GI dysfunction and changes to the GI tract’s microbiota in sepsis occur as part of an interactive sequence of feedback loops that control both local and systemic effects; the disrupted intestinal barrier becomes increasingly permeable to microbial products and live microbes, and the altered microbiota produces a unique environment for metabolism and immunity that exacerbates barrier repair and mucosal homeostasis disruption (Di Vincenzo et al., 2024). In sepsis, these loops are often intensified by infection-driven systemic inflammation, hemodynamic instability, vasopressor exposure, and broad-spectrum antibiotic pressure, which jointly accelerate barrier breakdown and ecological collapse. These reciprocities extend beyond the GI system to influence remote organs via circulating mediators, immune cells, and neurohormonal pathways. Understanding these bidirectional exchanges helps explain why patients with comparable pathogen loads and similar macro-hemodynamics can exhibit heterogeneous trajectories, including persistent organ dysfunction. In this section, we summarize sepsis-relevant mechanisms linking barrier disruption, microbiota alterations, and remote organ injury, and highlight points along this cascade that may be therapeutically targetable.

### 4.1 Mutual amplification between barrier disruption and dysbiosis

Following sepsis-associated microcirculatory injury and inflammatory signaling that compromises the integrity of the epithelium, the intestine becomes more and more of a “leaky” interface for intestinal contents (Chancharoenthana et al., 2023; Massier et al., 2021). The disassembly of tight junctions, apoptosis of epithelial cells, and the disruption of mucous layers create various pathways through which luminal substances will come into contact with the lamina propria. Host and immune cells residing within the lamina propria will encounter larger concentrations of microbial associated molecular patterns (MAMPs), including lipopolysaccharides, peptidoglycan, and flagellin, and damage-associated host molecules (Alpkvist, 2024). This sensing activates pattern recognition receptors and downstream signaling, increasing cytokines, chemokines, and proteolytic enzymes, which further injure epithelial/endothelial cells, degrade extracellular matrix, and impair microvascular regulation (Cicchinelli et al., 2024). At the tissue level, this will increase tissue damage as a result of additional damage to epithelial and endothelial cells, degradation of the extracellular matrix, and continued impairment of microvascular function.

In parallel, the dysbiotic microbiota that emerges in sepsis is selectively advantaged in this inflamed, oxygen-enriched, and antibiotic-pressured environment. In septic cohorts, reduced microbial diversity and expansion of hospital-adapted pathobionts are commonly observed and are often associated with secondary infection risk and worse outcomes, supporting the clinical relevance of this ecological shift. Facultative anaerobes and opportunistic pathogens can attach to exposed extracellular matrix and denuded epithelium, form biofilms at injured sites, and utilize host-derived nutrients released during tissue damage (Montanari et al., 2025). In contrast to the commensals, these facultative anaerobes and pathogens are producing metabolites that differ significantly in nature. While the commensals produce trophic short-chain fatty acid metabolites, the facultative anaerobes and pathogens produce metabolites that are either pro-inflammatory or toxic (Hays et al., 2024; Langfeld et al., 2021; Nabizadeh et al., 2023). Some of these metabolites may impair epithelial mitochondrial function and/or disrupt tight junction assembly, thereby further compromising barrier integrity. Other metabolites can shift local pH/redox conditions in ways that further favor facultative anaerobes/pathobionts, reinforcing their ecological dominance. Thus, barrier failure and dysbiosis form a self-perpetuating loop in sepsis: barrier injury promotes pathobiont expansion and altered metabolite profiles, and these microbiome changes, in turn, sustain inflammation and delay barrier repair.

### 4.2 Cross-organ axes: gut–liver, gut–lung, and gut–brain pathways

Disturbances of the gut microbiota in sepsis have consequences that can be communicated to the body’s internal organs through various structural and physiological paths. Due to its high proportion of blood supply coming from the portal vein (which drains the gut), the gut-liver path is particularly important as an example of how a disturbance of the gut can affect distant organs. When there is a loss of barrier function and when there is dysbiosis, it is easier for products from microbes and toxins produced by microbes, along with inflammatory mediators to travel through the portal circulation to the liver (Moura and Siqueira, 2025). As such, Kupffer cells and sinusoidal endothelial cells produce pro-inflammatory cytokines, chemokines and acute-phase proteins; hepatocytes alter their metabolic functions and detoxification pathways in order to manage the insult (Chen et al., 2024). Although the initial response of the liver is thought to help eliminate microbial products and protect the rest of the body’s circulatory system; over time, continuous exposure can lead to cholestasis, dysfunction of hepatocytes, and failure to properly convert endogenously produced compounds and drugs into non-toxic forms. These changes in the liver will then affect the gut by creating different bile acid compositions and flows that can further affect the gut’s microbial populations, motility, and barrier integrity, completing yet another loop.

The gut-lung axis is another significant route of communication between the gut and lungs during sepsis. Products of microbial metabolism and toxins can pass through the gut’s epithelium due to an increase in the gut’s permeability and the presence of dysbiosis. Once in the bloodstream, these products can eventually make their way to the pulmonary vasculature and alveolar space. Immune cells and endothelial cells within the lungs are responsive to these products, and their activation can contribute to further leakage of the vasculature, recruitment of neutrophils, and formation of micro-thrombi within the lung. Acute Lung Injury, or Acute Respiratory Distress Syndrome (ARDS), is a common complication of sepsis and is one of the most feared (Zhou and Liao, 2021).

## 5 Therapeutic modulation of the gut–microbiota axis in sepsis

The increasing understanding that the gut, along with its resident microbiota, can modulate sepsis pathophysiology has generated interest in therapeutic strategies that target the gut–microbiota–barrier axis. These approaches range from established ICU practices (e.g., early enteral nutrition and antibiotic stewardship) to emerging microbiota-directed interventions, including probiotics, prebiotics, symbiotic, postbiotics, fecal microbiota transplantation (FMT), and next-generation live biotherapeutics. Importantly, in sepsis the feasibility and efficacy of these interventions are strongly influenced by timing (early shock vs recovery), concurrent antibiotic exposure, hemodynamic stability and mesenteric perfusion, and the patient’s baseline microbiome and immune phenotype. Although these approaches share the objective of restoring gastrointestinal homeostasis, their mechanisms, evidence strength, and safety profiles differ substantially; therefore, patient selection and trial design are critical.

### 5.1 Early enteral nutrition

Enteral nutrition functions to modulate the interaction between the gut and the microbiome via the maintenance of the structural integrity of the mucosa, stimulation of digestive secretions; promotion of growth in the commensal flora; and the activation of neuroendocrine reflex mechanisms controlling intestinal motility and perfusion. Early enteral feeding may therefore be helpful in sustaining epithelial turnover and promoting the continued production of short chain fatty acids in sepsis where the progression of villous atrophy, disruption of tight junctions, and alterations in the microbiota are rapid (Moon et al., 2023).

However, there are significant challenges to the application of early enteral feeding in critically ill patients. The hemodynamic instability and high levels of vasopressors used in many critically ill patients raise concerns about the adequacy of mesenteric perfusion and the risk of intestinal ischemia, while ileus and delayed gastric emptying impede the ability of these patients to tolerate enteral feeding. Accordingly, in septic shock, enteral nutrition strategies should be individualized and dynamically titrated to perfusion status and gastrointestinal tolerance, rather than applied uniformly across all stages of illness.

### 5.2 Probiotics, prebiotics, synbiotics, and postbiotics

The goal of microbiota-directed biologics is to add back lost commensal bacteria, to reinstate metabolic function, and to modify an overactive immune response (Lou et al., 2023). However, the septic gut is a challenging site for colonization, as it is under constant antibiotic pressure; it is altered by bile acids; it has damaged epithelium; and it contains pro-inflammatory mediators that inhibit colonization of bacteria (Zahirović et al., 2025). Therefore, it can occur that while the bacteria are present in stool, they do not colonize the mucosa. This colonization failure underscores the need for alternative strategies that bypass the requirement for microbial engraftment—particularly postbiotics, defined as inanimate microbial cells, their components, or metabolites (e.g., short-chain fatty acids, bacteriocins, and cell wall fragments) that confer health benefits to the host. Unlike live probiotics, postbiotics are not affected by the hostile microenvironment of the septic gut (e.g., inflammation, antibiotic exposure, and epithelial damage) and can directly exert biological effects, such as enhancing intestinal barrier integrity, modulating immune responses, and suppressing pathogenic bacterial growth, without the risk of bacteremia/fungemia (Zheng et al., 2023). Additionally, there is significant variation between individuals, and therefore, this makes it difficult to predict the outcome in each individual. Safety of probiotics in patients who have severe immunosuppression and barrier damage is still of concern because there have been instances of bacteremia/fungemia from probiotics (Lejeune et al., 2025). Prebiotics may cause gas and distension, and postbiotics avoid the barrier to engraftment but must be used with caution when used in patients because of variability in the metabolism of the host. A most effective approach to using these products will likely be to characterize the type of dysbiosis (which functions rather than types of bacteria) that a patient needs to replace.

### 5.3 Selective digestive decontamination

Selective Digestive Decontamination (SDD) employs topical, nonabsorbed antibiotic agents plus short courses of systemic antibiotics for the purpose of suppressing pathogens with gram-negative rods while preserving the anaerobic commensal bacteria (Lou et al., 2023). Some intensive care units have reported a decrease in the number of cases of ventilator associated pneumonia and bloodstream infections.

However, SDD can substantially reshape the gut ecosystem, and it may not reliably preserve anaerobes. Ecological niches created by antibiotics may be colonized by resistant organisms or previously minor taxa, a risk heightened in septic patients already receiving systemic antimicrobials. Thus, SDD may reduce selected infections while simultaneously promoting antimicrobial resistance or further ecological disruption, underscoring the importance of local resistance epidemiology and stewardship frameworks when considering implementation.

### 5.4 FMT and next-generation live biotherapeutics

The goal of FMT is to rapidly replace a severely dysbiotic community with a diverse donor microbiota. While FMT is highly effective for recurrent C. difficile infection, several limitations constrain its application in sepsis, including safety and feasibility concerns (Yu et al., 2023). Even with donor screening, introducing a complex microbial community into an inflamed and permeable gut raises concerns about pathogen transmission and adverse inflammatory responses. In addition, concurrent antibiotics commonly used in sepsis may impair engraftment and durability of donor microbes.

Next-generation live biotherapeutics (NG-LBPs) aim to address several limitations of FMT by using standardized, well-characterized organisms or defined microbial consortia with consistent manufacturing and quality control. These approaches include (i) defined consortia designed to restore depleted functions (e.g., butyrate production, bile acid transformation), (ii) rationally selected strains targeting specific pathways (e.g., barrier-supporting or immunoregulatory signaling), and (iii) engineered bacteria that deliver therapeutic molecules or modulate inflammatory circuits. Compared with FMT, NG-LBPs offer greater controllability, reproducibility, and potentially lower risk of transmitting unwanted pathogens; however, in sepsis they still face key challenges such as antibiotic-associated engraftment failure, uncertain therapeutic windows, and safety considerations in barrier-compromised hosts. Future sepsis-oriented studies should therefore prioritize (a) mechanism-linked endpoints (barrier integrity and metabolite restoration), (b) staged administration strategies (post-antibiotic or recovery windows), and (c) rigorous safety monitoring.

### 5.5 Targeted metabolite and barrier-supporting therapies

Targeted interventions are designed to correct deficiencies in important microbial functions rather than alter the composition of the microbiota. The short chain fatty acids, particularly butyrate, have been shown to be important in maintaining the integrity of the tight junctions as well as mucus and regulating the immune system, therefore providing a potential mechanism to overcome the loss of these critical functions associated with dysbiosis (Roy et al., 2025). In addition, correcting bile acid signaling and providing enterocyte-targeted nutrients such as glutamine may support epithelial repair and restitution.

Barrier-supporting therapies should be discussed as a complementary category alongside metabolites, because epithelial integrity is a proximate determinant of microbial translocation in sepsis. Representative strategies include: (i) epithelial repair and proliferation signals (e.g., epidermal growth factor, EGF), (ii) modulation of cytoskeletal contraction and tight junction opening via myosin light-chain kinase (MLCK)–associated pathways, and (iii) tight junction–targeted approaches that stabilize claudins/occludin/ZO-1 localization and downstream regulators (e.g., RhoA/ROCK-related signaling). Additional supportive strategies include reinforcing the mucus layer and antimicrobial peptide defenses (via goblet/Paneth cell–supporting pathways), which may reduce direct epithelial exposure to luminal toxins and pathobionts.

Additional pharmacologic agents can be used to stimulate epithelial repair and/or the formation of tight junctions; however, they will be effective in an environment that is not toxic or inflammatory from the perspective of bacteria capable of producing toxins or inflammatory mediators. As has been seen with some metabolic supplements, overdosing could potentially support the growth of pathogens or disrupt the immune-metabolic homeostasis, highlighting the importance of carefully timed and dosed supplementation.

Additional pharmacologic agents can be used to stimulate epithelial repair and/or the formation of tight junctions; however, they will be effective in an environment that is not toxic or inflammatory from the perspective of bacteria capable of producing toxins or inflammatory mediators. As has been seen with some metabolic supplements, overdosing could potentially support the growth of pathogens or disrupt the immune-metabolic homeostasis, highlighting the importance of carefully timed and dosed supplementation. A comparative overview of the main gut-microbiota axis-targeted therapies discussed in this section is provided in **Table 2**.

**Table 2:** Comparison of gut-microbiota axis-targeted therapeutic approaches in sepsis.

Treatment method	Core mechanism of action	Clinical evidence grade	Main safety risks	Ideal patient phenotype
Early Enteral Nutrition (EN)	Maintains mucosal structural integrity; stimulates digestive secretions; promotes commensal flora growth; activates neuroendocrine reflexes regulating motility/perfusion.	Grade I-II Multiple RCTs and meta-analyses support early EN in critically ill patients, though direct sepsis-specific evidence varies.	Risk of intestinal ischemia if mesenteric perfusion inadequate; aspiration pneumonia; feeding intolerance/ileus.	Hemodynamically stable patients without severe ileus; those with preserved mesenteric perfusion; early phase of sepsis.
Probiotics/Prebiotics/Synbiotics/Postbiotics	Probiotics: Add live beneficial microbes. Prebiotics: Provide substrate for commensals. Synbiotics: Combine both. Postbiotics: Provide microbial components/metabolites without live bacteria.	Grade II-III Mixed evidence from RCTs; some show reduced infections/VAP, others show neutral effects. High heterogeneity in strains/doses.	Probiotics: Risk of bacteremia/fungemia in immunocompromised or barrier-damaged hosts. Prebiotics: Gas, distension, osmotic diarrhea. Postbiotics: Fewer colonization risks, but host metabolic variability affects response.	Patients with moderate dysbiosis and preserved barrier function; those not severely immunosuppressed; post-antibiotic phase for microbiota restoration.
Selective Digestive Decontamination (SDD)	Uses topical nonabsorbed + short-course systemic antibiotics to suppress Gram-negative pathogens while sparing anaerobes.	Grade I-II Strong evidence for reducing ventilator-associated pneumonia (VAP) and bloodstream infections in ICU settings, though sepsis-specific data are part of broader critical illness studies.	Potential selection for antibiotic-resistant organisms; further disruption of microbiota ecology; risk of Clostridioides difficile infection.	High-risk ICU patients anticipated to require prolonged mechanical ventilation; units with low baseline antibiotic resistance rates; as part of infection control bundles.
Fecal Microbiota Transplantation (FMT)	Rapidly introduces a diverse, healthy donor microbiota to replace dysbiotic community.	Grade III (experimental for sepsis) Established efficacy for recurrent C. difficile infection (Grade I). Limited case series/pilot studies in sepsis, no large RCTs.	Transmission of pathogens (even with screening); exacerbation of inflammation in permeable gut; poor engraftment due to concurrent antibiotics; procedural risks.	Refractory dysbiosis with recurrent infections post-antibiotics; specialized settings with strict donor screening and monitoring; possibly after antibiotic course completion.
Targeted Metabolites & Barrier-Support Therapies	Directly supplements deficient microbial metabolites (e.g., butyrate) to support tight junctions, mucus production, and immune regulation; provides nutrients (glutamine) for enterocyte repair.	Grade II-III Preclinical evidence strong; human trials in critical illness are emerging but limited. Some small RCTs show benefits in barrier markers.	Overdosing may support pathogen growth or disrupt homeostasis; variable host metabolism; insufficient data on optimal dosing/timing in sepsis.	Patients with documented metabolite deficiencies (e.g., low SCFAs); those with significant barrier disruption but controlled infection/inflammation; as adjunct to other therapies.
(e.g., SCFAs like butyrate, glutamine, bile acid modulators)				

### 5.6 Integrating gut-microbiota therapies into personalized sepsis care

Due to high inter-individual variability in dysbiosis severity, antibiotic exposure, barrier injury, and immune state, a “one size fits all” approach is unlikely to be effective for septic shock. A personalized strategy should incorporate biomarkers that reflect barrier integrity, microbial function, and systemic inflammation, and stratify patients by clinically actionable microbiome phenotypes (Piccioni et al., 2024). For example, patients with severe barrier disruption and high risk of translocation may preferentially benefit from barrier-stabilizing strategies, whereas those in recovery phases with persistent loss of obligate anaerobes may be better candidates for function-restoring approaches (e.g., targeted metabolites or defined consortia). Gut-directed therapies should be integrated into standard sepsis care alongside hemodynamic optimization, ventilatory strategies, and antimicrobial stewardship to create physiologic conditions that permit intestinal recovery and increase the likelihood of therapeutic benefit.

## 6 Limitations, challenges, and future directions

### 6.1 Clinical heterogeneity and treatment-related confounding

Although there has been a significant increase in research concerning gastrointestinal dysfunction and the gut-microbiota axis in sepsis, there remain numerous technical and conceptual difficulties that affect the reliability of the data currently available. In the case of clinical studies assessing gastrointestinal dysfunction in patients with sepsis, many studies have included large numbers of patients who were diagnosed with different types of infection, as well as a variety of comorbid conditions, and received various treatments for these conditions. Oftentimes, these studies also failed to utilize standardized and/or contemporary definitions of sepsis. The descriptions of gastrointestinal dysfunction in both clinical studies and experimental models of sepsis include inconsistent terminology and lack of valid methods for determining either barrier integrity or motility in response to sepsis. The presence of confounders related to concurrent treatments is widespread in septic patients. The majority of septic patients receive multiple systemic antibiotics throughout their illness, with many receiving repeated courses of antibiotics that significantly alter the composition of the microbiota. Other treatments including proton pump inhibitors, vasopressors, sedatives, opioids, and parenteral nutrition all impact gastrointestinal function and the ecological structure of the microbiota. Therefore, it is challenging to isolate the effect of sepsis itself from that of its treatments using observational study designs. Together, these sources of clinical heterogeneity (variable sepsis definitions, infection sources, baseline comorbidities, and ICU exposures) complicate between-study comparability and may partly explain neutral or conflicting results in gut-targeted trials.

Additionally, while randomized control trials designed to target the gut-microbiota axis may report neutral or conflicting findings because of the presence of treatment related confounders that overwhelm the treatment effects.

### 6.2 Sampling and sequencing limitations

Additional technical complications associated with microbiome research contribute to the difficulty in interpreting the results of such studies. Although the majority of studies examining the intestinal microbiota in response to sepsis have utilized 16S rRNA sequencing to evaluate the intestinal microbiota, this approach can only provide limited taxonomic resolution and little information regarding the function of the intestinal microbiota. Although a number of additional multi-omic technologies, including shotgun metagenomics, metatranscriptomics, and metabolomics, have recently been developed and applied to the study of the intestinal microbiota, the application of these technologies to cohorts of patients with sepsis is limited by factors related to the high costs of performing such analyses, sample instability, and analytic complexity. The use of different techniques for DNA extraction, different sequencing platforms, and different bioinformatics pipelines creates a considerable amount of variability between studies, making it difficult to reproduce the results of previous studies and to identify consistent microbial “signatures” of sepsis.

The use of indirect measures of the gut microbiota (e.g., stool samples) is an additional limitation of the sampling approach. While these samples can be useful surrogates for the gut microbiota, they do not accurately reflect the microbiota present on the mucosal surface (particularly in the small intestine), which likely plays a more critical role in the pathogenesis of sepsis. In addition, stool-based profiling may underrepresent mucosa-associated organisms and functions that directly interface with epithelial and immune compartments, limiting mechanistic inference. In most cases, direct sampling of the small intestine mucosa or lumen is impractical in critically ill patients due to their hemodynamic instability; thus, interactions between the epithelial cells and microbial communities in the gut are inferred indirectly rather than directly measured.

### 6.3 Challenges in causal inference

Additionally, because most studies have utilized a single time point for obtaining a biopsy of the intestinal microbiota (which was typically post-sepsis onset and subsequent to multiple interventions), it is very difficult to determine if the observed changes in the intestinal microbiota occurred prior to sepsis onset, drove the development of multi-organ failure associated with sepsis, or simply reflect the process of multi-organ failure associated with sepsis. This limitation introduces substantial ambiguity regarding directionality (cause vs consequence), as well as risks of reverse causation and residual confounding, particularly when key exposures (antibiotics, nutrition, vasopressors) evolve dynamically over time. Moreover, the temporal mismatch between host responses (e.g., cytokine surges, barrier breakdown) and microbiome measurements further complicates attribution of mechanistic pathways.

### 6.4 Translational barriers: safety, engraftment, timing, and personalization

Another area of focus is improving the mechanistic interpretability of interventional studies to better understand how they work. All trials assessing strategies for enteral nutrition, probiotics, synbiotics, FMT or metabolite supplementation should include both baseline and post-intervention assessments of the patient’s microbiota composition and barrier function as well as their clinical outcomes. This will allow investigators to determine if the intervention affects the targeted aspect of the gut-microbiota axis and, when, and to whom, these changes result in a clinical outcome. Beyond mechanistic readouts, translation is constrained by safety concerns in unstable patients (e.g., risk of bacteremia/fungemia with live biotherapeutics, pathogen transmission with FMT), variable engraftment under antibiotic pressure, and uncertainty regarding optimal therapeutic windows across sepsis stages. These challenges argue for individualized strategies guided by host–microbiome phenotypes rather than “one-size-fits-all” interventions.

Adaptive trial designs are possible, allowing investigators to modify either the inclusion criteria or the dosing (of an intervention) based on interim results from microbial analysis. These designs can potentially reduce the number of trials that are considered “negative” and mask potential benefit in certain subgroups. Additionally, ethical and practical considerations need to be addressed. Techniques used to collect samples and manipulate the microbiota are risky in unstable patients who have hemodynamic instability or are critical care patients. Intensivists, microbiologists, gastroenterologists, and methodologists will need to collaborate with each other to develop safe and minimally invasive methods for collecting samples and manipulating the microbiota in intensive care unit (ICU) settings. Where direct sampling is infeasible, minimally invasive sampling strategies and validated surrogate biomarkers of barrier function and microbial metabolism will be essential to support safe, scalable clinical implementation. Additionally, researchers could explore parallel areas of study in less acutely ill patients (e.g., sepsis survivors or high-risk individuals), and this type of research will provide information that is not limited by the problems associated with conducting research in unstable ICU patients.

### 6.5 Future directions: longitudinal cohorts, harmonized multi-omics, and mechanism-informed trials

The results of this research indicate a number of areas where additional research should occur. A primary area is in conducting longitudinal studies. These would involve repeated assessment of the microbiome, metabolome, and markers of gut barrier integrity during the time period of infection, through the acute phase of illness, into the convalescent phase (or full recovery) of illness to provide a better basis for making cause-and-effect interpretations, and to determine if there are transient disruptions vs. chronic, or dysbiotic states. The addition of standardized assessment of GI symptoms, an organ failure score, and detailed treatment data would greatly enhance the ability to make meaningful interpretation from these studies.

Another high priority is harmonization of methodologies used to collect samples, store them, sequence the microbes, and perform multi-omics analyses on the samples, along with developing bioinformatics workflows and reporting standards that can be applied consistently among studies. This would significantly reduce the technical variability of studies and enable researchers to compare findings among different studies in a meaningful way. A critical advancement in the field of gut health/microbiota will come from the integration of multi-omics analytical strategies including microbial taxonomy, microbial function, host gene expression, host protein levels, and immune phenotyping to move beyond descriptive correlations to a mechanistic understanding of how alterations in the gut microbiota result in disease pathology. Additional advanced statistical tools such as causal inference models and machine learning may assist in identifying stable microbial or metabolic patterns that predict disease course, or responsiveness to targeted treatments aimed at modifying the gut microbiota. Ultimately, a significant next step in the field will be the development of clinically useful composite phenotypic profiles of the gut-microbiota axis, which can combine measures of epithelial permeability, intestinal motility, splanchnic blood flow, microbial diversity, and key functional pathways to define patient populations most likely to benefit from particular treatments.

These phenotypes may also serve as inclusion/exclusion criteria for future clinical trials and to develop rapid bedside assays of selected microbial or host-based biomarkers to facilitate the use of these approaches. Future trials should be explicitly mechanism-informed, incorporating prespecified hypotheses, longitudinal sampling, and standardized endpoints to establish causal links and identify stage-specific therapeutic windows under real-world ICU exposures.

## 7 Conclusion

The bidirectional interplay between gastrointestinal (GI) dysfunction and gut microbial dysbiosis is a central driver of sepsis pathogenesis, transforming the GI tract from a passive target of systemic inflammation into an active effector that modulates immune responses, cross-organ communication, and clinical outcomes. This review integrates relevant evidence, indicating that microvascular perfusion deficiency, epithelial barrier disruption, and motor disorders caused by sepsis will disrupt the balance of intestinal microbiota. The dysregulated microbiota (characterized by reduced diversity, decreased beneficial anaerobic bacteria, and increased pathogenic symbiotic bacteria) exacerbate gastrointestinal dysfunction through pro-inflammatory metabolites and impaired barrier repair dysfunction. This self-reinforcing cycle extends beyond the GI tract, mediating injury through gut-liver, gut-lung, and gut-brain axes, and contributes to the heterogeneous clinical trajectories of septic patients.

Transforming these insights into clinical practice requires specifically regulating the axis of the intestinal microbiota. Key strategies include early enteral nutrition to maintain mucosal integrity, biological agents targeting the microbiota (such as postbiotics as a safe alternative in inflammatory bowel diseases, rather than probiotics), and fecal microbiota transplantation to treat refractory microbiota dysbiosis. The key to success lies in personalized approaches - guided by biomarkers of barrier function (such as the integrity of tight junctions) and microbial metabolism (such as the levels of short-chain fatty acids), and with attention to timing, as intervention measures may vary depending on the stage of sepsis (early and recovery period).

In summary, integrating the physiology of the GI tract and microbiome science into sepsis research transforms our understanding of sepsis from a multi-organ disease to a host-microbe systems disorder. By addressing current limitations and prioritizing mechanistic and translational research, we can transition from gut-tolerant to gut-conscious care, ultimately improving outcomes for septic patients.
